# Hydrochloric acid-enhanced radiofrequency ablation for treating a large hepatocellular carcinoma with spontaneous rapture: a case report

**DOI:** 10.1186/s40880-016-0161-8

**Published:** 2017-01-07

**Authors:** Jin-Hua Huang, John N. Morelli, Fei Ai, Ru-Hai Zou, Yang-Kui Gu, Fei Gao, Tian-Qi Zhang, Wang Yao, Xiong-Ying Jiang, Yan-Yang Zhang

**Affiliations:** 1 Department of Minimally Invasive Interventional Therapy, Sun Yat-sen University Cancer Center, State Key Laboratory of Oncology in South China, Collaborative Innovation Center for Cancer Medicine, Guangzhou, 510060 Guangdong P. R. China; 2 Department of Interventional Radiology, St. John’s Medical Center, Tulsa, OK 74131 USA; 3 Department of Oncologic Interventional Therapy, The First Affiliated Hospital, Sun Yat-sen University, Guangzhou, 510080 Guangdong P. R. China; 4 Department of Radiology Intervention, Sun Yat-sen Memorial Hospital, Sun Yat-sen University, Guangzhou, 510120 Guangdong P. R. China; 5 Department of Intervention, The Third Affiliated Hospital, Sun Yat-sen University, Guangzhou, 510630 Guangdong P. R. China

**Keywords:** Hydrochloric acid, Radiofrequency ablation, Spontaneous rupture, Hepatocellular carcinoma

## Abstract

**Background:**

A ruptured hepatocellular carcinoma (HCC) is often fatal. In addition to surgery and transarterial embolization, radiofrequency ablation (RFA) might be another option for treating a ruptured HCC. Unfortunately, conventional RFA has a limited ablation zone; as such, it is rarely used to treat ruptured tumors.

**Case presentation:**

This case was a 60-year-old man who had a large, ruptured HCC in which hydrochloric acid (HCl)-enhanced RFA successfully controlled the bleeding and made the tumor completely necrotic.

**Conclusion:**

Considering the effectiveness of HCl-enhanced RFA in achieving hemostasis and tumor ablation, it might be a new option for treating large, ruptured HCCs.

## Background

Liver injuries are common and often result in abdominal pain, bleeding, hypotension, and other conditions requiring immediate attention. Hepatic hemorrhage most commonly occurs because of blunt trauma or the spontaneous rupture of hepatic neoplasms, including hepatocellular carcinoma (HCC), adenoma, focal nodular hyperplasia, and hemangioma.

 In Asia and Africa, the most common neoplastic cause of hepatic hemorrhage is HCC, owing to the high prevalence of the disease [1]. The occurrence rate of spontaneously ruptured HCC ranges from 3% to 14.5%; a ruptured HCC with intraperitoneal hemorrhage is a life-threatening complication with a 30-day hospital mortality between 31% and 67% [2, 3]. Several factors are associated with hemorrhagic rupture of HCC, including trauma, large tumor size, vascular invasion, multiple nodules, prior transarterial chemoembolization (TACE), portal venous hypertension, and vascular injury [3, 4]. Hepatic hemorrhage related to a ruptured HCC can rapidly cause hemodynamic instability that requires emergency treatment.

Treatment options for ruptured hemorrhagic HCC include packing, plication, emergent hepatic artery ligation, hepatectomy, transarterial embolization (TAE), and radiofrequency ablation (RFA) [3, 5, 6]. In some cases, TAE and surgery are not effective. Moreover, a 3-cm ablative zone produced by a single RFA probe or a 5-cm ablative zone obtained with a cluster of probes is not sufficient to ablate a large tumor. However, hydrochloric acid (HCl)-enhanced RFA has a much larger ablation zone, making it potentially suitable for controlling bleeding from a large, ruptured HCC.

## Case report

**Fig. 1:**
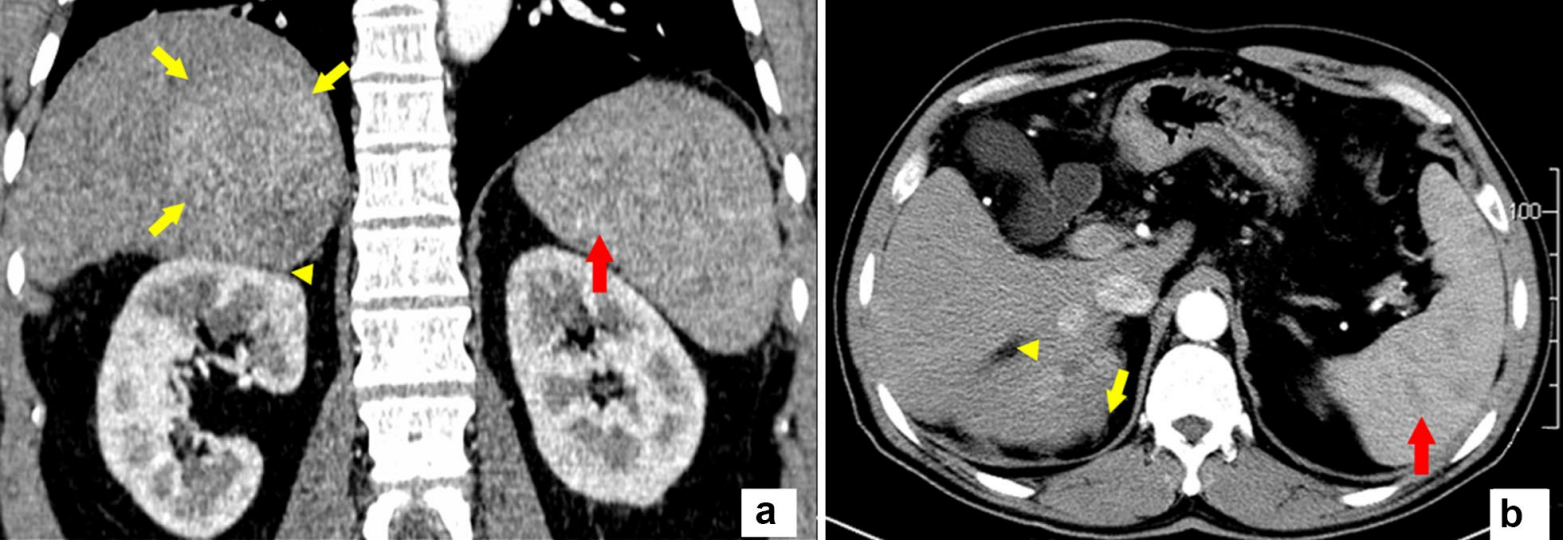
The initial computed tomography (CT) images of the patient with large hepatocellular carcinoma. The coronal **a** and axil **b** CT images in the arterial phase show a tumor (5.8 cm × 7.6 cm) occupying liver segments VI–VII (*yellow arrows*) with multifocal lesions. The inferior portion of the tumor protrudes through the liver contour adjacent to the ipsilateral renal fascia (*yellow arrowhead*). Splenomegaly can be seen as well (*red arrow*)

**Fig. 2:**
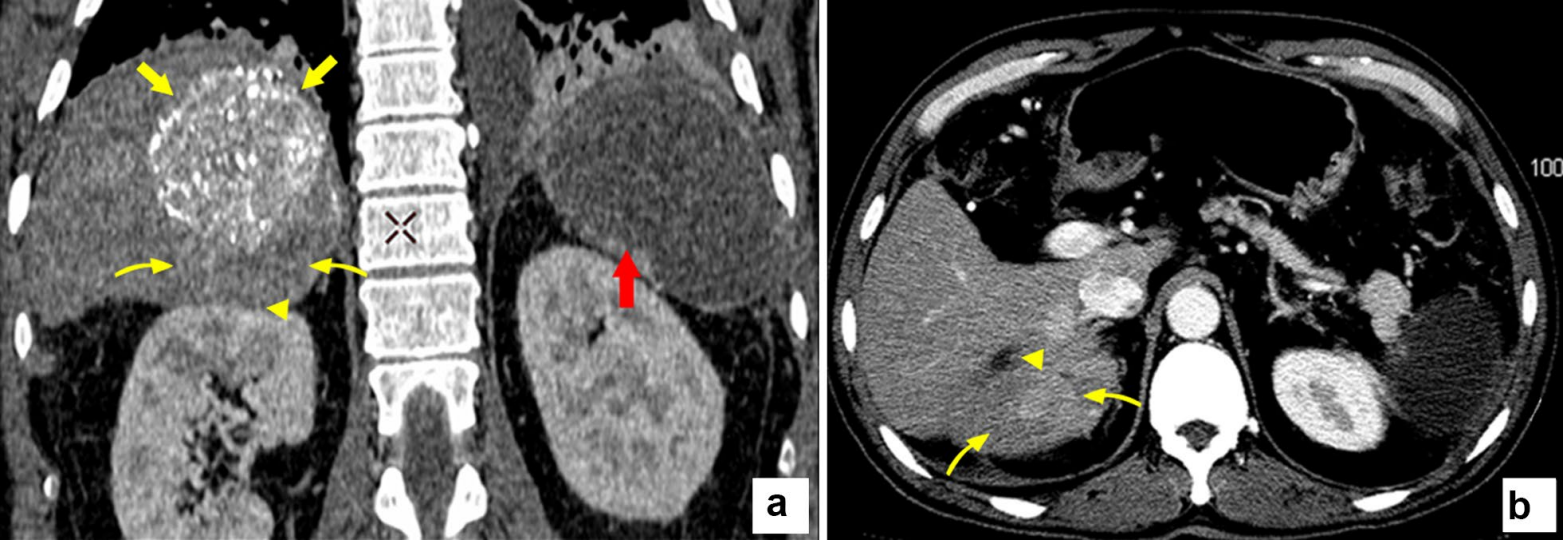
The follow-up CT images after transarterial chemoembolization (TACE). The coronal **a** and axil **b** CT images show the tumor with inhomogeneous deposition of lipiodol, the compact upper portion (*yellow arrow*) and the lower portion (*yellow curved arrow*) without lipiodol deposition which was still enhanced in the arterial phase. The protruded tumor was located adjacent to the upper polar of the right kidney (*yellow arrowhead*). The spleen was inactive after partial embolization (*red arrow*)

On December 13, 2013, a 60-year-old man presented at Sun Yat-sen University Cancer Center with a diagnosis of HCC (Barcelona Clinic Liver Cancer stage B; Fig. 1), severe cirrhosis, and splenomegaly. On January 9, 2014, because of his impaired liver function (evaluated as Child-Pugh B) and low platelet count, he underwent TACE and a partial spleen embolization (PSE). A subsequent contrast-enhanced computed tomography (CT) scan taken on February 27, 2014 revealed a tumor with compact deposition in the upper portion and suboptimal deposition in the lower portion (Fig. 2). Moreover, his platelet count increased from 45.0 × 10^9^/L at baseline to 115.0 × 10^9^/L after PSE, which is in the normal range, and his carbohydrate antigen concentration decreased from 216.1 to 111.9 U/mL. As the patient had Child-Pugh B liver function and moderate anemia, we therefore thought that he could tolerate a second TACE.

**Fig. 3:**
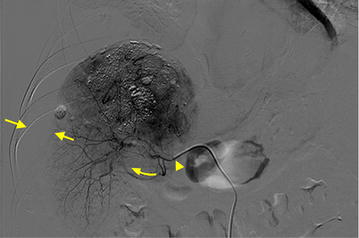
The digital subtraction angiography image of the emergent transarterial embolization for arresting bleeding. No lipiodol deposition is observed in the upper portion of hepatocellular carcinoma (*curved arrow*). The feeding arterial vessel is difficult to trace (*arrowhead*). The crumpled hepatic parenchyma is separated from the abdominal wall by the hemoperitoneum (*twin arrows*)

On March 15, 2014, after pre-TACE workup, the patient experienced sudden abdominal pain following a hard defecation. Based on obvious signs of peritoneal irritation and uncoagulated hemorrhagic fluid in the perihepatic region obtained under ultrasound-guided abdominal paracentesis, a diagnosis of a spontaneously ruptured HCC was confirmed. An emergent TAE was performed by the physicians on duty. Digital subtraction angiography (DSA) showed that a large amount of intraperitoneal hemorrhage had pushed the liver parenchyma away from the abdominal wall (Fig. 3). Unfortunately, the patient became hemodynamically unstable during the next 12 h, and increasing ascites and active bleeding were confirmed by ultrasound. His liver function deteriorated to Child-Pugh C, making surgery inappropriate. Considering several reports of ruptured HCC being controlled by emergent RFA and our experience with ablation-expanding, HCl-enhanced RFA, we decided to try to control his bleeding with HCl-enhanced RFA [6, 7].

Written informed consent to the procedure and consent to publish were obtained from the patient and his family. The HCl-enhanced RFA procedure was approved by the Clinical Research Ethics Committee of Sun Yat-sen Cancer Center.

**Fig. 4:**
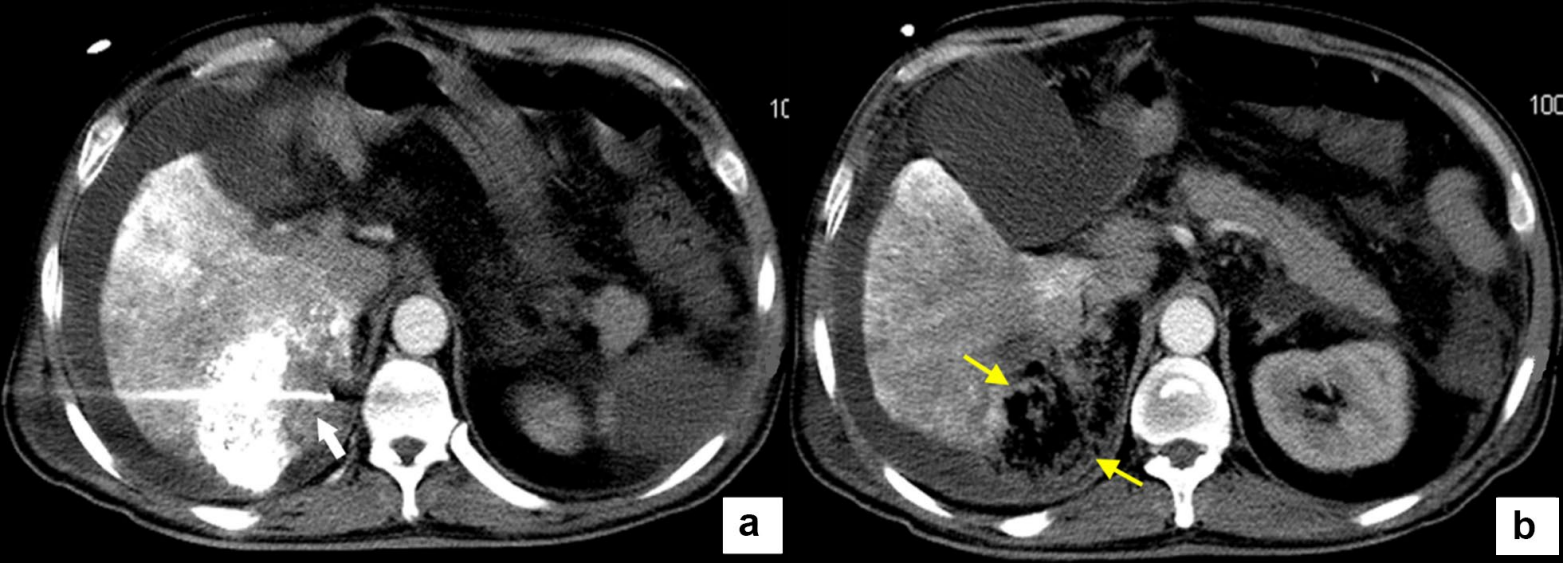
The CT images of the hydrochloric acid (HCl)-enhanced radiofrequency ablation (RFA) procedure. **a** The RFA electrode (*arrow*) is inserted into the portion without lipiodol deposition. **b** The loose necrotic HCC lesion after HCl-enhanced RFA procedure hangs on the renal region (*twin arrows*)

A large ablative zone can be achieved with HCl-enhanced RFA by constantly infusing HCl at a rate of 0.2 mL/min and simultaneously delivering radiofrequency (RF) energy with an RF generator (Model 1500X, AngioDynamics, RITA Medical Systems, Queensbury, NY, USA) coupled with a monopolar perfusion electrode (UniBlate, RITA Medical Systems). We used 10% HCl (Huayi Medical Auxiliary Materials Manufacturing Co., Ltd, Chengdu, Sichuan, China) as the perfusate, which was diffused in the center of the tumor. HCl is thought to increase the ionic concentration around the electrode and delay charring of the surrounding tissue [7]. With the patient under local anesthesia, HCl-enhanced RFA was applied for 100 min with a constant stable output power of 100 W and a temperature of 103 °C to the potential bleeding site, which was probably the portion of the protruding liver contour without any lipiodol deposition (Fig. 4).

**Fig. 5:**
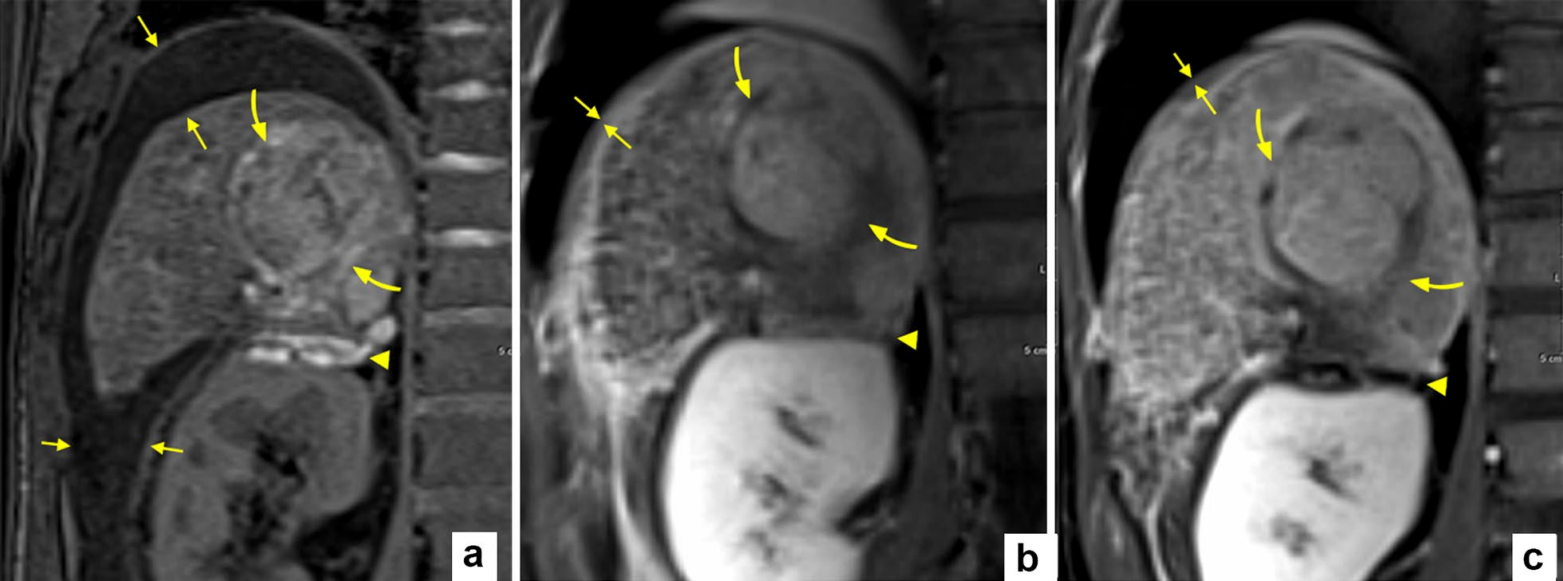
The follow-up magnetic resonance images (MRI). The coronal MRI at 1 (**a)**, 6 (**b)**, and 9 months (**c)** after HCl-enhanced RFA show reduced lesion size and a satisfactory tumor capsule beyond the hepatic contour (*curved arrow*). The gradually distinct margin of the upper renal fascia is also visible (*arrowhead*). Perihepatic and perirenal ascites are decreased (*twin arrows*)

After CT-guided, HCl-enhanced RFA, the patient’s vital signs returned to normal within 24 h. The peritoneal drainage fluid changed from hemorrhagic dark red to light yellow, which confirmed control of the bleeding. Additional conservative treatments were administered during follow-up. Moreover, magnetic resonance imaging performed 1, 6, and 9 months later showed a necrotic tumor with little liquid surrounding the hepatic capsule (Fig. 5). By the end of October 2015, the patient had been alive for 16 months and had an acceptable quality of life, with Child-Pugh B liver function.

## Discussion

Spontaneous tumor rupture is a potential consequence of HCC and results in a high mortality [3]. The most common mechanisms for hemoperitoneum in HCC rupture are tumor pseudo-capsule disruption and tearing of a parasitic feeding artery. Kanematsu et al. [8] hypothesized that several features visible on CT images portend HCC rupture: a large tumor size, an area of protruded tumor, and a large extent of contact between the tumor and the liver surface. Consistent with this hypothesis, the large HCC in this case eventually ruptured.

Hemostasis is the primary consideration in HCC rupture, and adequate bleeding controlling is vital to patient prognosis. As treatment opinions regarding ruptured HCC have evolved and as new clinical technologies have been adopted, treatment strategies for ruptured HCC have become increasingly diverse, including various conservative, surgical, and minimally invasive measurements. In 1965, Ong et al. [9] advocated packing the ruptured liver as an efficient method to control hemorrhage; then, in 1972, they advocated ligating the hepatic artery. However, with simple packing, they found a high rate of re-bleeding. Moreover, in some cases in the 1970s, direct plication by overlying the capsule was determined to achieve hemostasis [8]. Because the results were disappointing, though, given the friability of HCC, this method is now seldom used. Although emergent hepatectomy remains the most effective treatment for ruptured HCC, it accounts for 7% of the hospital mortality [3].

With the increasing application of interventional techniques in the last 20 years, TAE emerged as another procedure that could potentially achieve hemostasis in a ruptured HCC, having a success rate between 53% and 100% and resulting in a lower 30-day hospital mortality (0%–37%) than those of surgery [5]. The advantages of TAE over surgery are that it is minimally invasive and directly occludes the feeding vessels, which means that surgery can be avoided in patients at high risk.

Recently, several studies reported successful hemostasis using RFA [6, 10]. In addition, Chueng et al. [1] found that RFA is safe and effective for achieving hemostasis in ruptured HCC with satisfactory survival outcomes, and therefore they proposed RFA as a first-line option for treating a ruptured HCC. However, needle placement with laparoscopic RFA is less accurate than that with image-guided percutaneous RFA, which has particular advantages for treating lesions within the deep hepatic parenchyma.

In the present case, because our patient had severe cirrhosis, he could not tolerate surgery. Moreover, a failed TAE impaired his hepatic function. As Leung et al. [11] reported, non-super-selective TAE as regional therapy is harmful to patients. They thought that there was typically only one opportunity to achieve hemostasis with TAE, and that more than one attempt would harm the normal hepatic parenchyma.

When the tumors are large or peripherally located, extrahepatic collateral arteries commonly supply HCCs [12]. In our case, both enhanced CT scanning and DSA revealed a filling defect on the lower portion of the tumor. Thus, considering the location of the non-enhanced tumor, collateral vessels supplying this portion were probably branches of the adrenal artery.

After TAE failed to restore hemostasis, and considering both the patient’s overall condition and the results of our previous clinical studies with HCl-enhanced RFA, we determined that CT-guided RFA was a reasonable treatment option. A prior PSE had increased the patient’s platelet count to the level on which coagulation was adequate to proceed with RFA [13]. However, currently, the maximum ablative zone obtainable with conventional monopolar RFA is 3 cm in diameter. Thus, in this case, the 7-cm diameter tumor would be difficult to ablate in one session. Additionally, our previous experiments showed that infusing diluted HCl instead of natural saline into the ablation zone during RFA with monopolar perfusion electrodes could enlarge the ablation size (mean ± standard deviation) from 3.49 ± 0.07 to 6.85 ± 0.32 cm under the same power and time settings (30 W and 30 min) [14]. Given these considerations and the patient’s large hepatic mass, we decided to use HCl-enhanced RFA instead of natural saline-infused RFA. In our case, HCl-enhanced RFA damaged neither the nearby duodenum nor the renal fascia. This result may relate to the progressive decrease in temperature from the center to the periphery and to the insulating effects of the fibrous fascia.

In contrast to normal saline, HCl functions as a chemical ablation agent and is capable of producing an ablative lesion itself [15]. Furthermore, HCl is a strong electrolyte whose conductivity is about three times that of natural saline, which can increase the conductivity of RF energy. Thus, when HCl is instilled around the RF electrode, the RF energy expands from a needle shape into a spherical shape. This enlarged spherical volume increases contact area between the RF source and the tissue intended for ablation. In turn, this increased contact area transfers and sustains RF energy much longer (100 min in this case) than normal saline, lengthening the charred area and prolonging the delivery of energy, thus creating a larger ablation zone than any other current RFA method. Therefore, our success in treating this ruptured HCC suggests that a treatment pattern of HCl-enhanced RFA followed by TAE or combined with surgery could be considered in the future.

As in the present case, for patients with a cirrhotic liver, HCC is often associated with survival of less than 30 days [3]. However, our patient has survived for 16 months. Treatment with HCl-enhanced RFA achieved immediate and total hemostasis and completely ablated the large, bleeding tumor. In addition, as a regional therapy, HCl-enhanced RFA resulted in well-preserved liver function; thus, the patient could recuperate rapidly.

In conclusion, HCl-enhanced RFA appears to be a safe and effective treatment for controlling bleeding after spontaneous rupture of large HCC. However, further research is necessary to confirm our findings.
